# Serotonin improves glucose metabolism by Serotonylation of the small GTPase Rab4 in L6 skeletal muscle cells

**DOI:** 10.1186/s13098-016-0201-1

**Published:** 2017-01-03

**Authors:** Ramona Al-Zoairy, Michael T. Pedrini, Mohammad Imran Khan, Julia Engl, Alexander Tschoner, Christoph Ebenbichler, Gerhard Gstraunthaler, Karin Salzmann, Rania Bakry, Andreas Niederwanger

**Affiliations:** 1 Department of Internal Medicine I, Medical University of Innsbruck, Innsbruck, Austria; 2 Section of Physiology, Medical University of Innsbruck, Innsbruck, Austria; 3 Institute of Analytical Chemistry and Radiochemistry, Leopold-Franzens University Innsbruck, Innsbruck, Austria

**Keywords:** Serotonin, GLUT4, Rab4, L6 skeletal muscle cells, Glucose uptake, Serotonylation

## Abstract

**Background:**

Serotonin (5-HT) improves insulin sensitivity and glucose metabolism, however, the underlying molecular mechanism has remained elusive. Previous studies suggest that 5-HT can activate intracellular small GTPases directly by covalent binding, a process termed serotonylation. Activated small GTPases have been associated with increased GLUT4 translocation to the cell membrane. Therefore, we investigated whether serotonylation of small GTPases may be involved in improving Insulin sensitivity and glucose metabolism.

**Methods:**

Using fully differentiated L6 rat skeletal muscle cells, we studied the effect of 5-HT in the absence or presence of insulin on glycogen synthesis, glucose uptake and GLUT4 translocation. To prove our L6 model we additionally performed preliminary experiments in C2C12 murine skeletal muscle cells.

**Results:**

Incubation with 5-HT led to an increase in deoxyglucose uptake in a concentration-dependent fashion. Accordingly, GLUT4 translocation to the cell membrane and glycogen content were increased. These effects of 5-HT on Glucose metabolism could be augmented by co-incubation with insulin and blunted by co incubation of 5-HT with monodansylcadaverine, an inhibitor of protein serotonylation. In accordance with this observation, incubation with 5-HT resulted in serotonylation of a protein with a molecular weight of approximately 25 kDa. We identified this protein as the small GTPase Rab4, the activity of which has been shown to be stimulated by both insulin signalling and serotonylation.

**Conclusion:**

Our data suggest that 5-HT elicits its beneficial effects on Glucose metabolism through serotonylation of Rab4, which likely represents the converging point between the insulin and the 5-HT signalling cascades.

## Background

Serotonin (5-hydroxytryptamine, 5-HT), the biogenic amine of the amino acid tryptophan, affects a broad range of behavioural, cognitive and physiological functions. 5-HT action is mediated by the binding of 5-HT to 5-HT-receptors that are divided into seven distinct classes (5-HT1 to 5-HT7) based on their structural and functional characteristics [1]. Medication that affects 5-HT metabolism is known to alter glucose homeostasis including antidepressants such as some selective 5-HT reuptake inhibitors (SSRIs), which increase 5-HT levels in the synaptic cleft, improve insulin sensitivity and glucose metabolism [2]. However, some second generation antipsychotics (SGAs) that antagonize the 5-HT2A-receptor (5-HT2AR) and inhibit the phosphoinositol-3 kinase (PI3 K)-dependent insulin signalling pathway [3] cause weight gain and insulin resistance [4], suggesting a link between serotonin and the PI3 K pathway. Supporting these findings, more recent studies have shown that some 5-HT2AR gene polymorphisms are associated with the development of obesity and deterioration of glucose homeostasis. Accordingly, patients harbouring these polymorphisms are more likely to develop diabetes under SGA-therapy [5, 6].

The underlying molecular mechanisms causing the improvement in insulin sensitivity and glucose metabolism by 5-HT and the connection between the serotonin and the PI3 K pathways are not fully understood.

Recently, several studies showed a novel mechanism of protein activation by covalent binding of 5-HT to a number of intracellular proteins after entering the cell through the 5-HT-reuptake transporter (SERT). This process is catalysed by transglutaminases and also involves the extracellular activation of 5-HT2AR to increase intracellular Ca2+ -levels, which are needed for the activation of these Ca2+ -dependent transglutaminases. One group of proteins activated by serotonylation are intracellular small GTPases. Paulman et al. showed in 2009 that serotonylation of small Rab-GTPases activates these proteins in pancreatic beta cells, promoting glucose-mediated insulin secretion [7]. Additionally, this group showed that tryptophan hydroxylase 1 knockout mice, which lack the enzyme needed to produce 5-HT, develop diabetes [8]. In support of these data, other research groups found that transglutaminase 2 knockout mice also become diabetic [9].

Activated small GTPases have been shown to regulate many steps in intracellular trafficking, such as vesicle formation, movement and membrane fusion [10]. Insulin-responsive tissues, such as skeletal muscle and adipose tissue, express several Rab and Rho isozymes. These activated small GTPases have been associated with GLUT4 translocation to the cell membrane, a process linked to the PI3 K-dependent insulin signalling pathway [11].

The aim of this study was to investigate whether the activation of Rab- and/or Rho-GTPases by serotonylation could represent the mechanism by which 5-HT increases GLUT4 translocation to the cell membrane, thus, increasing glucose transport and restoring glucose homeostasis. Here, we provide evidence that serotonylation of the small GTPase Rab4 represents the link between the PI3 K-dependent insulin and the 5-HT signalling cascades.

## Methods

### Materials

Amyloglucosidase and all inorganic reagents (mHT, 5-HT, MDC, pargyline) were from Sigma (St. Louis, MO, USA). The ECL-kit was from Amersham Biosciences (Buckinghamshire, UK). The [14C]-molecular weight marker, 2-deoxy-[1-3H]-glucose and 5-[1.2–3H(N)]-5-HT were from Perkin-Elmer (Boston, MA, USA). The 4–15% linear-gradient mini gels were from Bio-Rad (Hercules, CA, USA). Anti-Rab4 and the Rab- and Rho-GTPases sampler kits were from Cell Signaling Technology (Beverly, MA, USA). Anti-SERT, anti-Rho, anti-GLUT4 antibodies and the anti-pan-cadherin antibody were from Abcam (Cambridge, UK). The 5-HT-antibody was from AbDSerotec (Duesseldorf, Germany). The L6 rat skeletal muscle cell line, as well as the C2C12 cell line, the CaCo2 cell line and the PMC cell line were obtained from ATCC (Manassas, VA, USA). α-MEM, Eagle’s MEM and RPMI 1640 were purchased from Sigma (St. Louis, MO, USA), FCS from Biochrom AG (Berlin, Germany), BCS and fatty acid/insulin-free BSA from Sigma (St. Louis, MO, USA).

### Cell culture

L6 and C2C12 skeletal muscle cells—were cultured at 37 °C with 5% CO2 and used up to the ninth passage for the L6 cells. C2C12 were used following the second passage. Cells were grown to confluency in α-MEM containing 10% FCS and then switched to the same media containing 2% FCS for differentiation. Experiments were performed with fully differentiated myotubes 12–14 days post-confluency for the L6 and on the 6th day post-confluency for the C2C12 cells.

PBMC cells—were cultured at 37 °C with 5% CO2. Cells were grown in RPMI 1640 containing 10% BCS.

CaCo2 cells—were cultured at 37 °C with 5% CO2. Cells were grown in Eagle’s MEM containing 10% FCS.

Incubation experiments—One day prior to the experiments, the medium was replaced with starvation medium containing serum-free α-MEM with 0.25% insulin-free BSA. The next morning, cells were incubated with 5-HT (in the concentration 0.01; 0.1; 1; 10 and/or 100 µM as indicated in the respective figure legends), mHT (in the concentration 0.01; 0.1; 1; 10 and/or 100 µM as indicated in the respective figure legends), 25 µM MDC, 1 µM pargyline in the absence or presence of 100 nM insulin for the lengths of time indicated in the respective figure legends.

Then, cells were lysed and analysed for glucose uptake, glycogen content, GLUT4 translocation and protein content.

### Deoxyglucose uptake and calculation of the apparent Vmax and Km

After starvation, cells were incubated in HBS for 1 h in the absence and presence of methyl-5-HT (mHT), 5-HT, monodansylcadaverine (MDC), pargyline and insulin at 37 °C. Then, 2-deoxyglucose uptake was measured as previously described by our group [12]. Vmax and Km values were calculated using non-linear least square fitting (MatLab, version 6.1, The MathWorks, Natick, MA, USA).

### Glycogen content

After starvation, cells were incubated with the respective reagents in the absence or presence of insulin for 1 h. Glycogen content was then analysed by the method of [13] with minor modifications as previously described by our group [14].

Western blot analysis—Following starvation overnight, L6 cells and C2C12 cells were incubated for 1 h in the absence and presence of 5-HT, MDC, pargyline and insulin as indicated in the respective figure legends. All subsequent steps for Western blot analysis were performed as previously described [14]. As a loading control, an antibody against β-actin was used.

Membrane protein isolation for the analysis of GLUT4 translocation - This experiment was performed in the absence or presence of 5-HT, MDC, pargyline and insulin following overnight starvation. Cells were washed with PBS and collected in buffer 1 (5 mM Tris–HCL pH 7.4, 2 mM EDTA and protease inhibitors). Lysis was performed by sonification, and lysates were centrifuged for 15 min at 500×*g*. The supernatant of this centrifugation step was again centrifuged at 45.000×*g* for 15 min. The resulting pellet was washed in buffer 1 and resuspended in buffer 2 (75 mM Tris–HCl pH 7.4, 12.5 mM MgCl2, 5 mM EDTA). All subsequent steps for Western blot analysis were performed as previously described [14]. To control for loading, an antibody against pan-cadherin was used.

### 5-HT uptake assay

Uptake measurements were performed with overnight starved cells at increasing 5-HT concentrations as indicated in the respective figure legend. Krebs–Ringer-HEPES (KRH)-buffer was used as an incubation media. As a reaction buffer, KRH-buffer containing 3[H]-5-HT was used. The cells were immediately washed with KRH-buffer and incubated with the reaction buffer at room temperature for 1, 12, 60 and 360 s at the concentrations indicated [15]. Cells were then solubilized in 0.05 M NaOH, and samples were analysed in a scintillation counter. Vmax and Km values were calculated using non-linear least square fitting in a Michaelis–Menten model (MatLab, version 6.1, The MathWorks, Natick, MA, USA).

AnnexinV-Apoptosis Assay—Apoptosis/necrosis in L6 cells was evaluated by Annexin V/PI (propidium iodide) double staining. We could not detect any necrosis/apoptosis by AnnexinV/PI-staining with 5-HT, mHT, MDC and pargyline incubations.

### Data analysis

ANOVA for repeated measurements was performed for the factors insulin and mHT, 5-HT, MDC and pargyline. When univariate analysis revealed significant differences, post hoc comparisons were made using Bonferroni’s least significant difference method. Otherwise, univariate results were stated. All values were expressed as mean ± S.D., and statistical significance was accepted as P < 0.05.

## Results

### Expression of SERT and cellular uptake of 5-HT

**Fig. 1:**
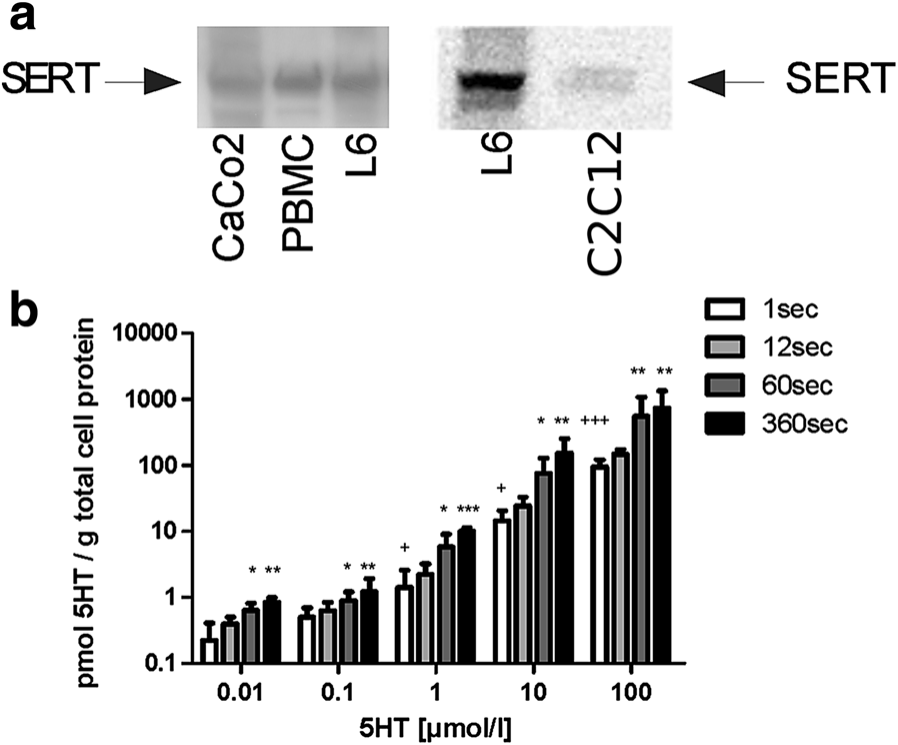
**a** *SERT*-*expression in L6 and C2C12 skeletal muscle cells.* Western blot analysis of total cell lysates using a SERT-specific antibody. Expression of SERT was compared between CaCo2 cells (human colon carcinoma cell line) and PBMC cells (monocyte cell line) two cell lines for which SERT-expression has been previously described [16, 17], and L6 cells (N = 6) and C2C12 cells respectively (N = 2). **b** *Concentration*- *and time*-*dependent uptake of 5*-*HT into L6 cells using radioactive 5*-*HT.* L6 cells were incubated for increasing incubation times (1, 12, 60 and 360 s) and increasing [^3^H]-labelled 5-HT concentrations (0.01; 0.1; 1; 10 and 100 µM) and analysed for 5-HT uptake. Bars represent the mean ± S.D. for three experiments performed in triplicates. Significant differences are indicated as ^*^P < 0.05 ^**^P < 0.01 ^***^P < 0.001 for comparisons to the condition at 1 s incubation time and at the same 5-HT concentration, and ^+^P < 0.05 ^+++^P < 0.001 for comparisons to condition at 1 s incubation time and at the 5-HT concentration of 0.01 µM

First, we studied the expression of the 5-HT re-uptake transporter SERT and the cellular uptake of 5-HT. Western blot analysis revealed that SERT is expressed on the cell membrane of L6 and C2C12 skeletal muscle cells (Fig. 1). Expression of SERT on L6 cells was compared with CaCo2 cells (human colon carcinoma cell line) and PBMC cells (monocyte cell line) two cell lines for which SERT-expression has been previously described [16, 17] (Fig. 1a).

Moreover, 5-HT uptake into L6 skeletal muscle cells occurred in a time- and concentration-dependent fashion (Fig. 1b).

### 5-HT and mHT increase glucose uptake and glycogen content in a concentration-dependent fashion

**Fig. 2:**
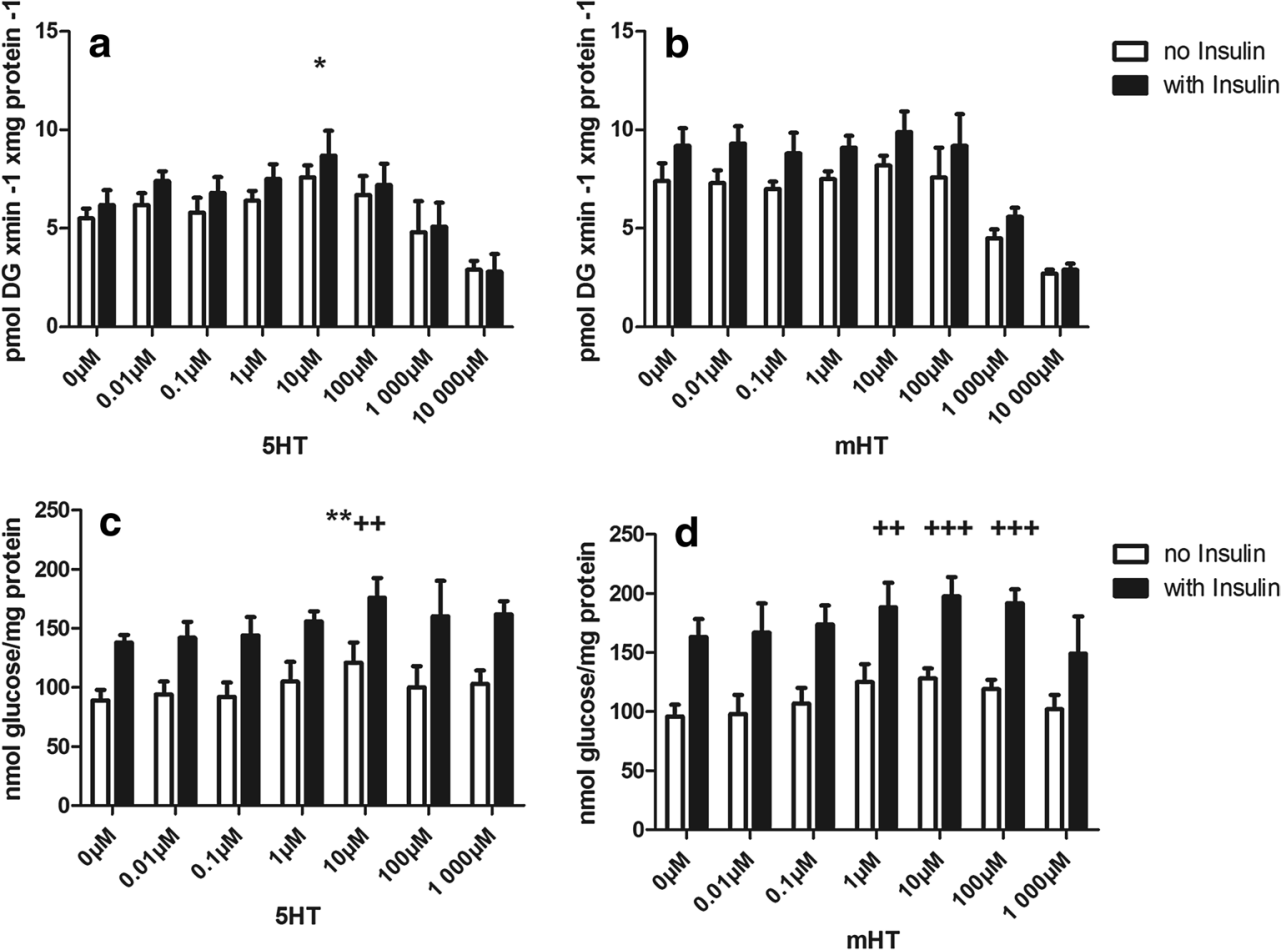
**a**, **b** *Analysis of glucose uptake with increasing concentrations of 5*-*HT* (**a**) o*r mHT* (**b**). L6 cells were incubated with increasing concentrations of either 5-HT or mHT in the presence (*solid bar*) or absence (*empty bar*) of 100 nM insulin for 1 h. Significant differences are indicated as ^*^P < 0.05 for comparison to the condition at 0 nM of 5-HT in the absence of insulin. (N ≥ 3 in triplicate). **c**, **d** *Analysis of glycogen content with increasing concentrations of 5*-*HT (* **c** *) or mHT* (**d**). L6 cells were incubated with increasing concentrations of either 5-HT or mHT in the presence (*solid bar*) or absence (*empty bar*) of 100 nM insulin for 3 h. Significant differences are indicated as ^**^P < 0.01 for comparison to the condition at 0 nM of 5-HT in the absence of insulin and ^++^P < 0.01 or ^+++^P < 0.001 for comparison to the condition at 0 nM of 5-HT or mHT in the presence of insulin (N ≥ 3 in triplicate)

In these experiments, we used L6 cells to study the effect of both 5-HT and methyl-5-HT (mHT), a more specific ligand for the 5-HT2AR than 5-HT, on glucose uptake (Fig. 2a, b) and glycogen content (Fig. 2 c, d); 5-HT induced a significant increase in glucose uptake, however, only with 5-HT at a concentration of 10 µM in the absence of insulin (Fig. 2a). Glycogen content was significantly increased at a concentration of 10 µM 5-HT both in the absence and presence of insulin (Fig. 2c). With mHT, significance was reached at 1 µM but only in the presence of insulin (Fig. 2d).

Interestingly, the effects of 5-HT and mHT on glucose uptake and glycogen content were augmented in the presence of insulin. To characterize the kinetics of 5-HT-induced glucose uptake, a Michaelis–Menten kinetic model using non-linear least square fitting was used. With this model, we observed additive mechanisms for insulin and 5-HT action on glucose uptake.

**Fig. 3:**
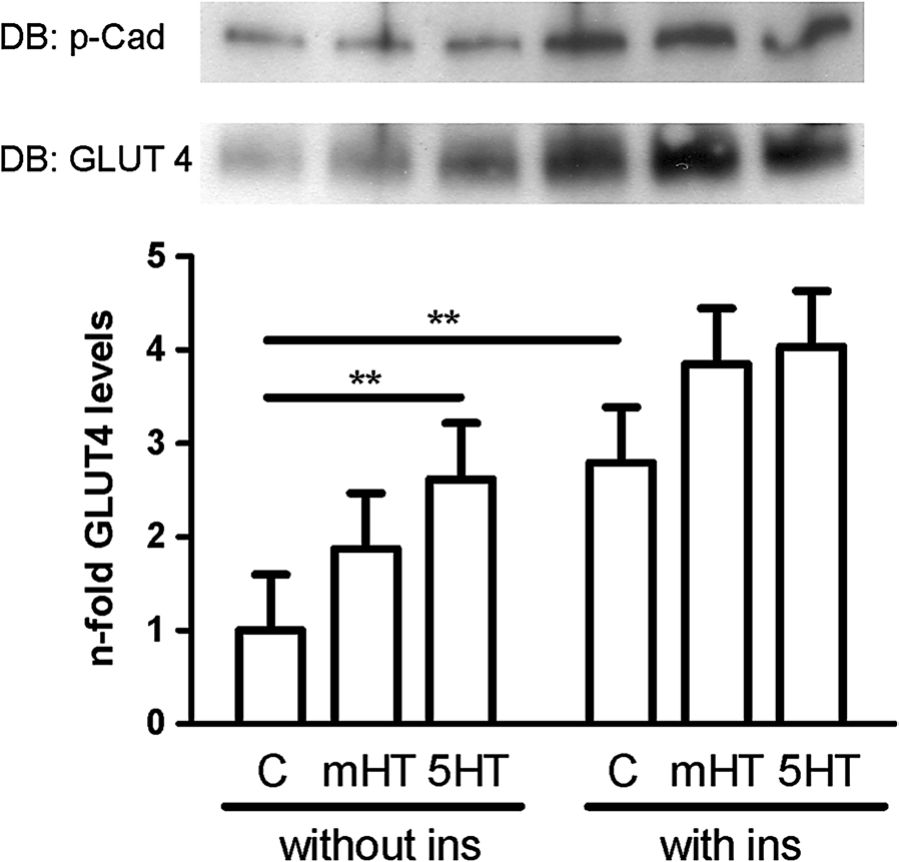
*5*-*HT increases GLUT*-*4 translocation.* L6 cells were incubated with 10 µM 5-HT or mHT in the absence or presence of 100 nM insulin for 1 h. After cell lysis, the lysates were used for membrane protein isolation followed by immunoblotting (IB) with an antibody to GLUT4. Detection of pan-cadherin (p-Cad) was used as a loading control. Significant differences are indicated as ^**^P < 0.01. (N = 4 in duplicate)

As shown in Fig. 3, insulin elicited the expected increase in GLUT4 translocation. In the absence of insulin, GLUT4 translocation was augmented with 5-HT incubation, indicating that increased GLUT4 translocation represents the basis of the observed positive effects of 5-HT on glucose metabolism.

As 5-HT had a more pronounced and consistent effect on glucose metabolism than mHT, only 5-HT at a concentration of 10 µM was used for all the following experiments.

### Effect of pargyline and monodansylcadaverine on 5-HT induced increases in glucose uptake, GLUT4 translocation and glycogen content

**Fig. 4:**
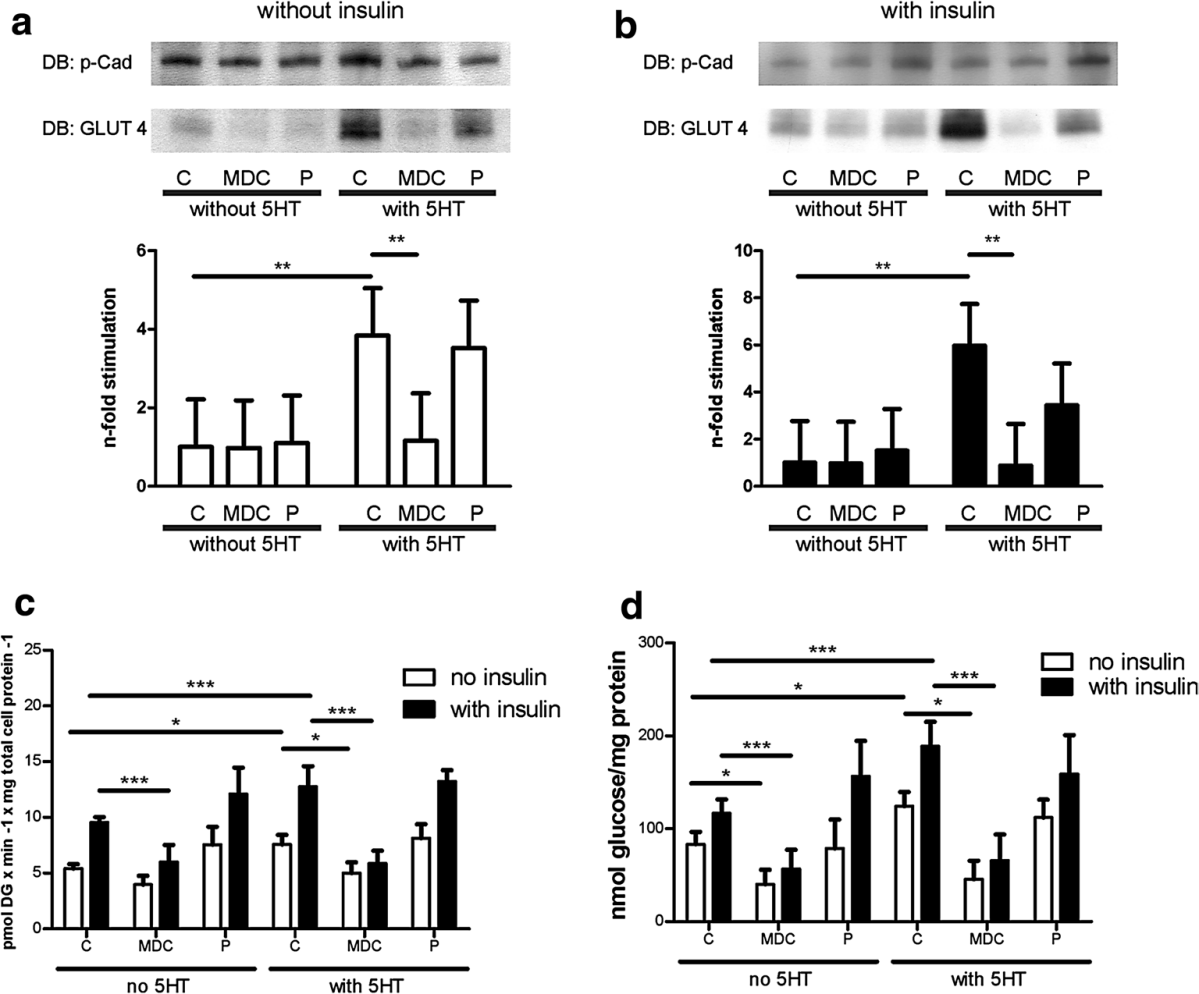
A and B. *5*-*HT*-*induced GLUT*-*4 translocation is reduced by MDC.* L6 cells were incubated without (**a**) or with (**b**) 100 nM insulin for 1 h. Incubations were performed with 25 µM MDC, 1 µM pargyline (P) or without either of these substances (C), in the absence or presence of 10 µM 5-HT. Cell lysates were used for membrane protein isolation followed by immunoblotting (IB) with an antibody against GLUT4 (N = 3). Detection of pan-cadherin (p-Cad) was used as a loading control. Significant differences are indicated as ^**^P < 0.01. **c** *5*-*HT*-*induced glucose uptake is reduced by MDC.* L6 cells were incubated without or with 100 nM insulin for 1 h. Incubations were performed with 25 µM MDC, 1 µM pargyline (P) or without either of these substances (C), both in the absence or presence of 10 µM 5-HT. Significant differences are indicated as ^*^P < 0.05, ^***^P < 0.001 (N = 3 in triplicate). **d** *5*-*HT*-induced glycogen content is reduced by *MDC.* L6 cells were incubated without or with 100 nM insulin for 3 h. Incubations were performed with 25 µM MDC, 1 µM pargyline (P) or without either of these substances (C), in the absence or presence of 10 µM 5-HT. Significant differences are indicated as ^*^P < 0.05, ^***^P < 0.001 (N = 3 in triplicate)

To determine whether 5-HT itself or its metabolites were responsible for the positive effects on glucose metabolism in L6 cells, the degradation of 5-HT was blocked with pargyline, a substance that inhibits the enzymatic activity of MAO. As shown in Fig. 4, pargyline did not diminish the 5-HT-induced increase of GLUT4-translocation (Fig. 4a, b), glucose uptake (Fig. 4c) or glycogen content (Fig. 4d).

To determine whether the effects of 5-HT were mediated by serotonylation of intracellular proteins, we used L6 cells and co-incubated 5-HT with monodansylcadaverine (MDC), a drug known to inhibit protein-serotonylation by blocking transglutaminases. MDC incubation at a concentration of 25 µM blunted the 5-HT-induced increase of GLUT4 translocation (Fig. 4a, b), glucose uptake (Fig. 4c) and glycogen content (Fig. 4d), both in the absence and presence of insulin.

### 5-HT induces serotonylation of several intracellular proteins

**Fig. 5:**
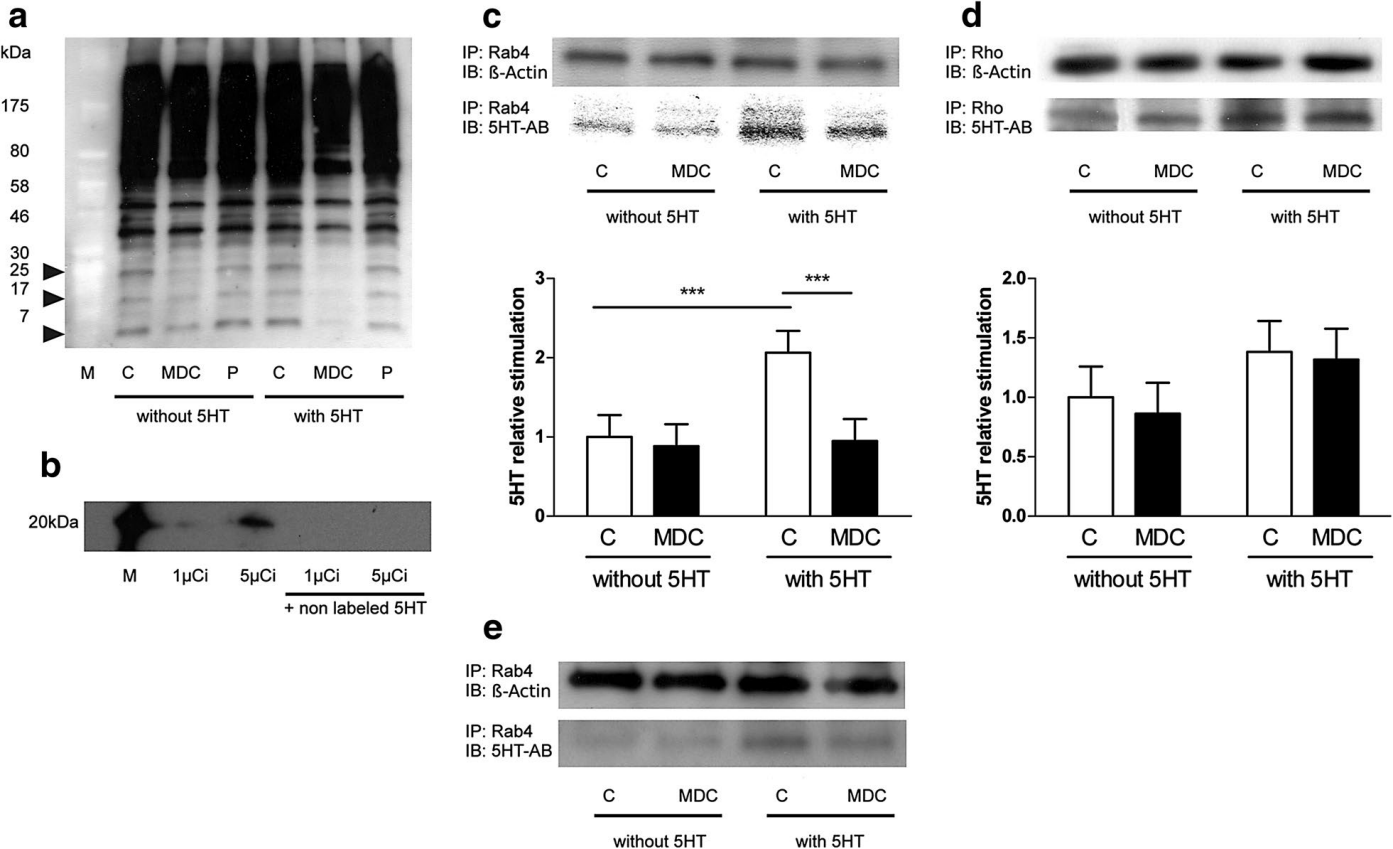
***a*** *Western blot analysis of total cell lysates using a 5*-*HT*-*specific antibody.* L6 cells were incubated with 25 µM MDC, 1 µM pargyline (P) or without either of these substances (C), in the absence or presence of 10 µM *5*-*HT* for 1 h. After cell lysis, *direct blotting* was performed with a 5-HT-specific antibody (N = 3). **b** *SDS gel analysis of total cell lysates after incubation with radioactive 5*-*HT.* L6 cells were incubated with [^3^H]-labelled 5-HT at 1 and 5 µCi for 1 h, then cells were lysed, and lysates were subjected to SDS gel analysis. Experiments were performed either in the absence or presence of non-labelled *5*-*HT* at a concentration of 10 µM (N = 4). *M*, radiolabelled molecular weight marker**. c** *Analysis of Rab4 serotonylation using a 5*-*HT*-*specific antibody.* L6 cells were incubated in the absence or presence of 10 µM 5-HT with or without MDC for 1 h, and lysed in lysis buffer. An aliquot of the lysates was subjected to SDS-PAGE for *direct blotting* (DB) with an antibody against Rab4, and the remaining lysate was used for immunoprecipitation (IP) with an antibody against Rab4 followed by immunoblotting (IB) with an antibody against beta-actin (ß-Actin) as a loading control and 5-HT respectively. The *bar graph* shows the quantification of serotonylated Rab4. *Bars* represent the mean ± S.D. for four independent experiments, with significant differences indicated as ^***^P < 0.001, C, Control. **d** *Analysis of Rho serotonylation using a 5*-*HT*-*specific antibody.* L6 cells were incubated in the absence or presence of 10 µM 5-HT with or without MDC for 1 h, and cells lysed in lysis buffer. An aliquot of the lysates was subjected to SDS-PAGE for direct blotting (DB) with an antibody against Rho, and the remaining lysate was used for immunoprecipitation (IP) with an antibody against Rho followed by immunoblotting (IB) with an antibody against beta-actin (ß-Actin) as a loading control and 5-HT respectively. The bar graph shows the quantification of serotonylated Rho. Bars represent the mean ± S.D. for four independent experiments. C, Control. **e** *Analysis of Rab4 serotonylation using a 5*-*HT*-*specific antibody.* C2C12 cells were incubated in the absence or presence of 10 µM 5-HT with or without MDC for 1 h, and lysed in lysis buffer. An aliquot of the lysates was subjected to SDS-PAGE for direct blotting (DB) with an antibody against Rab4, and the remaining lysate was used for immunoprecipitation (IP) with an antibody against Rab4 followed by immunoblotting (IB) with an antibody against beta-actin (ß-Actin) as a loading control and 5-HT respectively

To study whether 5-HT affects the serotonylation of intracellular proteins, we performed Western blot analyses with a 5-HT-specific antibody both in the absence and presence of 5-HT using L6 cells. As shown in Fig. 5a, direct blotting of cell lysates revealed several bands at various molecular weights. However, the signal of only three of these bands at molecular weights ranging from 5 to 25 kDa could be blunted by MDC incubation suggesting serotonylation of the corresponding proteins. L6 cells incubated with 3[H]-labelled 5-HT followed by SDS gel analyses of the cell lysates revealed a single band at a molecular weight slightly above 20 kDa (Fig. 5b). Incubation in the presence of non-labelled 5-HT abrogated this signal.

### 5-HT induces serotonylation of Rab4

To identify the band at a molecular weight slightly above 20 kDa, we performed immunoprecipitations with anti-phospho-serine, anti-phospho-threonine and anti-phospho-tyrosine antibodies, and immunoblotted with an antibody against 5-HT using L6 cells. These experiments showed no bands. Subsequently, we incubated L6 cells with 3[H]-labelled 5-HT and performed immunoprecipitations with anti-phospho-serine, anti-phospho-threonine and anti-phospho-tyrosine antibodies. Again, no bands were detectable (data not shown).

Next, we performed immunoprecipitations using L6 cells with antibodies against Rab4 and Rho proteins, both known to have molecular weights of approximately 25 kDa and to be serotonylated. Western blotting of the precipitates using a 5-HT-specific antibody was performed in the absence and presence of 5-HT. These experiments revealed that 5-HT increases the intensity of the band upon precipitation with the Rab4 antibody, and the strength of this signal could be reduced by co-incubation with MDC (Fig. 5c). To prove our L6 model we repeated the immunoprecipitation experiment with Rab4 using C2C12 murine cells. These experiments found again that 5-HT increases the intensity of the band upon precipitation with the Rab4 antibody, and the strength of this signal could be reduced by co-incubation with MDC (Fig. 5e). In contrast, 5-HT incubation yielded no differences in the signal strength of the bands precipitated with the Rho antibody (Fig. 5d).

**Fig. 6:**
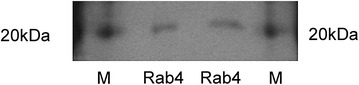
*Analysis of Rab4 serotonylation using radioactive 5*-*HT*. L6 cells were incubated in the presence of [^3^H]-labelled 5-HT at 5 µCi. After cell lysis, immunoprecipitation using a *Rab4*-antibody followed by SDS-PAGE was performed. *M*, radiolabelled molecular weight marker

To confirm that the band at approximately 25 kDa corresponds to Rab4, cells were incubated with 3[H]-labelled 5-HT followed by immunoprecipitation of the cell lysate with an antibody against Rab4. As shown in Fig. 6, a signal could be detected at the expected molecular weight of Rab4.

## Discussion

**Fig. 7:**
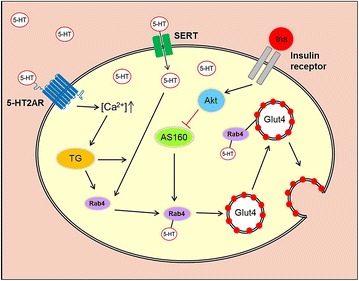
*Our proposed model of 5*-*HT*-*induced translocation of GLUT4 vesicles to the cell membrane of 5*-*HT*-*stimulated skeletal muscle cells (based on previous research performed by* Paulmann et al. [7] ]: Initially, extracellular 5-HT enters the cell via the 5-HT re-uptake transporter (SERT) while Ca2 + influx increases via activation of the 5-HT2A-receptor (5-HT2AR) by 5-HT binding. Among other functions, Ca2 + activates the transglutaminase (TG) which serotonylates a variety of intracellular proteins. In skeletal muscle cells, Rab4, which plays an important role in the translocation of GLUT4 to the cell membrane, becomes serotonylated and, thereby, activated. Consequently, glucose uptake into the cell increases. Activation of the insulin receptor resulting in inhibition of AKT-substrate 160 (AS160) also leads to the activation of Rab4 proteins indirectly

As mentioned in the introduction section, accumulating evidence indicates that 5-HT exerts a positive effect on whole body glucose homeostasis [2, 4]. However, the underlying molecular mechanisms are not fully understood. Here, we present evidence that transglutaminase-induced serotonylation of Rab4 leading to an activation of this protein represents a mechanism by which 5-HT improves glucose homeostasis in skeletal muscle cells (Fig. 7).

After showing that SERT is expressed on the cell membrane of our models L6 rat skeletal muscle cells and additionally in C2C12 murine skeletal muscle cells (Fig. 1a) and that L6 cells are able to take up 5-HT in a dose- and time-dependent manner (Fig. 1b), we demonstrated that 5-HT is able to increase glucose uptake (Fig. 2a), glycogen content (Fig. 2c) and GLUT4 translocation to the cell membrane (Fig. 3) in the absence of insulin. These effects of 5-HT on glucose uptake (Fig. 2b), glycogen content (Fig. 2d) and GLUT 4 translocation (Fig. 3) were augmented in the presence of insulin.

Several studies have shown that small GTPases can be activated by serotonylation [7, 8, 18]. Rab and Rho especially seem to be involved in intracellular insulin action and affect insulin-mediated GLUT4 translocation [11]. Although only shown in adipocytes so far, there is evidence that, of the Rab-family members, Rab4 in particular plays a key role in insulin-mediated cellular processes, including GLUT4 vesicle trafficking [19–21].

These independent observations, led us to hypothesize that serotonylation of Rab and/or Rho proteins might represent the mechanism underlying the 5-HT-induced improvements in glucose metabolism. Therefore, we investigated whether serotonylation is responsible for the 5-HT effects on glucose metabolism in L6 cells using MDC, an inhibitor of transglutaminases. MDC abolished the 5-HT-induced effects on GLUT4 translocation (Fig. 4a, b), glucose uptake (Fig. 4c) and glycogen content (Fig. 4d), supporting our hypothesis that 5-HT-induced serotonylation of intracellular proteins plays an important role in these processes. In contrast, pargyline did not diminish the 5-HT-induced increase of GLUT4 translocation (Fig. 4a, b), glucose uptake (Fig. 4c) and glycogen content (Fig. 4d), indicating that 5-HT itself, and not its metabolites, exerts the positive effects on glucose metabolism.

In the next step, we decided to use a 5-HT specific antibody to further test our hypothesis. This antibody detected numerous bands, however, with only three of these bands, ranging in size from 5 to 25 kDa, were down-regulated by MDC (Fig. 6a). Incubating the cells with 3[H]-labelled 5-HT, we could detect only one band slightly above 20 kDa (Fig. 7). Incubation with additional non-labelled 5-HT abrogated this signal, indicating that it competitively replaced the 3[H]-labelled 5-HT (Fig. 6b). To identify this band, we performed immunoprecipitations with antibodies against phospho-serine-, phospho-threonine- and phospho-tyrosine kinase in radioactive and non-radioactive settings. As these experiments yielded no positive results (data not shown), we used antibodies against Rho and Rab proteins, both known to be activated through serotonylation [7, 8, 18] and also known to play roles in GLUT4 trafficking [11, 19–21]. Using antibodies against the Rho proteins Cdc42, Phospho-Rac1/cdc42, Rac1/2/3, RhoA, RhoB and RhoC serotonylation was not detectable. However, we detected serotonylation following immunoprecipitation with a pan-Rho-antibody. Based on our observations that the band corresponding to the Rho protein was not affected by MDC and 5-HT, we concluded that Rho-serotonylation had no major role in this process (Fig. 6d). Using antibodies against the Rab proteins Rab4, Rab5, Rab9 and Rab11 only Rab4 immunoprecipitation gave a band upon immunoblotting with a 5-HT specific antibody, the signal of which was down-regulated by MDC and up-regulated by 5-HT, suggesting that Rab4 is activated by transglutaminase-dependent serotonylation (Fig. 6c).

We cannot exclude that other serotonylated proteins may contribute to the positive effects of 5-HT on glucose metabolism because MDC inhibits serotonylation of all proteins. Furthermore, the involvement of other Rab- or Rho-isoforms in addition to those examined cannot be excluded. Therefore, the impact of these serotonylated proteins on the regulation of cellular processes needs to be further investigated. Also proteins involved in cell contraction, such as actin, were reported to be activated by serotonylation in smooth muscle cells [22]. Because actin also participates in GLUT4 exocytosis and counteracts Rab- and Rho-GTPases, this protein may be of particular interest and will therefore be the issue of future experiments.

The prevalence of the metabolic syndrome and its long term consequences, such as cardiovascular diseases and type 2 diabetes mellitus, is increased in patients with depression and schizophrenic disorders, and the prevalence of depression is nearly twice as high in patients with type 2 diabetes as in healthy subjects [23, 24]. Considering that type 2 diabetes is the fourth leading cause of death in developed countries and is the leading cause of blindness, end stage renal disease and lower extremity amputations, and confers a two to four times greater risk of heart disease and stroke, the present study is of relevance for a considerable part of the world’s population [25, 26].

This study focused upon skeletal muscle cells as skeletal muscle is the predominant site of insulin-mediated glucose uptake and skeletal muscle insulin resistance is considered to be the primary defect in type 2 diabetes [27]. Furthermore, L6 rat skeletal muscle cells were shown to express the 5-HT2AR [28] and Rab4 [29], and this cell line is a well-established cell culture model for studying GLUT4 trafficking [30]. Nevertheless, use of this cell line restricts the translation of our results to human physiology. First, this cell line is derived from rats, and therefore species-specific differences must be considered. Second, these cells were stimulated by a concentration of human insulin 100-fold above normal postprandial insulin levels in humans, although the 5-HT concentrations used were comparable to the 5-HT levels found in platelet-poor human plasma [31]. Therefore, we believe that Rab4 serotonylation contributes to the effects of serotonergic agents on body glucose homeostasis in humans.

## Conclusion

In summary, our data reveal a molecular mechanism by which 5-HT improves glucose metabolism in skeletal muscle. Our data suggest a link between the 5-HT and the PI3 K insulin signalling pathway because insulin can also activate Rab4 via AS160 [32] (Fig. 7). Future studies should be designed to elucidate a possible role of serotonylation of Rab4 in the development of type 2 diabetes and to characterize other potential protein targets for serotonylation as the basis for 5-HT action. Our findings may represent the basis for future preventive and therapeutic strategies to overcome antipsychotic drug-induced insulin resistance and diabetes.

## Abbreviations

serotonin: 5-hydroxytryptamine, 5-HT
SSRIs: selective-serotonin-reuptake-inhibitors
MAO: monoaminooxidase
SGAs: second generation antipsychotics
5-HT2A-receptor: 5-HT2AR
SERT: serotonin reuptake transporter
GTPase: guanosine triphosphate hydrolyzing and binding protein
GLUT4: glucose transporter 4
mHT: methyl-5-HT
P: pargyline
MDC: monodansylcadaverine
AS160: AKT-substrate 160
